# Beneficial Effects of an 8-Week, Very Low Carbohydrate Diet Intervention on Obese Subjects

**DOI:** 10.1155/2013/760804

**Published:** 2013-03-14

**Authors:** Yunjuan Gu, Haoyong Yu, Yuehua Li, Xiaojing Ma, Junxi Lu, Weihui Yu, Yunfeng Xiao, Yuqian Bao, Weiping Jia

**Affiliations:** ^1^Department of Endocrinology and Metabolism, Shanghai Jiao Tong University Affiliated Sixth People's Hospital, Shanghai Diabetes Institute, Shanghai Clinical Centre of Diabetes, Shanghai 200233, China; ^2^Shanghai Key Laboratory of Diabetes Mellitus, Shanghai 200233, China; ^3^Department of Endocrinology and Metabolism, Affiliated Hospital of Nantong University, Nantong 226001, Jiangsu Province, China; ^4^Department of Radiology, Shanghai Jiao Tong University Affiliated Sixth People's Hospital, Shanghai 200233, China; ^5^Department of Endocrinology and Metabolism, Affiliated Hospital of Nantong University, Nantong, Jiangsu Province, China

## Abstract

*Aim*. To investigate the effects of weight loss during an 8-week very low carbohydrate diet (VLCD) on improvement of metabolic parameters, adipose distribution and body composition, and insulin resistance and sensitivity in Chinese obese subjects. *Methods*. Fifty-three healthy obese volunteers were given an 8-week VLCD. The outcomes were changes in anthropometry, body composition, metabolic profile, abdominal fat distribution, liver fat percent (LFP), and insulin resistance and sensitivity. *Results*. A total of 46 (86.8%) obese subjects completed the study. The VLCD caused a weight loss of −8.7 ± 0.6 kg (mean ± standard error (SE), *P* < 0.0001) combined with a significant improvement of metabolic profile. In both male and female, nonesterified fatty acid (NEFA) significantly decreased (−166.2 ± 47.6 **μ**mol/L, *P* = 0.001) and **β**-hydroxybutyric acid (BHA) increased (0.15 ± 0.06 mmol/L, *P* = 0.004) after eight weeks of VLCD intervention. The significant reductions in subcutaneous fat area (SFA), visceral fat area (VFA), and LFP were −66.5 ± 7.9 cm^2^, −35.3 ± 3.9 cm^2^, and −16.4 ± 2.4%, respectively (all *P* values *P* < 0.0001). HOMA IR and HOMA **β** significantly decreased while whole body insulin sensitivity index (WBISI) increased (all *P* values *P* < 0.001). *Conclusion*. Eight weeks of VLCD was an effective intervention in obese subjects. These beneficial effects may be associated with enhanced hepatic and whole-body lipolysis and oxidation.

## 1. Introduction

In the past decades, the prevalence of overweight and obesity has increased rapidly worldwide. Obesity is a major risk factor for several chronic diseases such as type 2 diabetes mellitus, cardiovascular disease, and some kinds of cancer [1]. Because of the severe comorbidities of obesity, people attempt to lose weight through several alternative diets. Recently, there has been a resurgence of interest in VLCD as a means of weight loss and metabolic improvements. Evidence from clinical studies and meta-analyses suggested that VLCD can decrease body weight, improve metabolic parameters, insulin resistance/sensitivity, and nonalcoholic fatty liver disease (NAFLD) [2]. However, the effect of VLCD on *β* cell function and insulin sensitivity is still controversial. Svendsen et al. reported that when ten obese women lost 11% of their baseline weight after an 8-week VLCD diet, their subcutaneous and intra-abdominal fatty tissue significantly decreased, but HOMA IR increased and peripheral insulin sensitivity was unaltered [3]. Malandrucco et al. observed that the metabolic profile of 14 severely obese patients with type 2 diabetes with a 7-day VLCD intervention were improved markedly primarily due to the amelioration of *β* cell function (disposition index increased), but with no change in insulin sensitivity [4]. Viljanen et al. found that after 16 obese subjects underwent a 6-week VLCD, visceral adipose and abdominal subcutaneous fat decreased while whole body insulin sensitivity increased, but adipose tissue regional glucose uptake remained unchanged [5]. Therefore, it is necessary to further assess the beneficial effect of VLCD, especially its impact on *β* cell function and insulin sensitivity.

It has been reported that the amount of carbohydrate intake affects blood glucose and insulin levels and even metabolic consequences [6]. However, in traditional Chinese dietary, carbohydrate contributes more than half of the total daily energy consumption. The method of VLCD to lose weight is less popular in Chinese comparing with that in westerns. Therefore, this is the first time to investigate the beneficial effects of VLCD on weight loss, improvements of body composition, insulin sensitivity and resistant, and plasma metabolic parameters in Chinese obese subjects. 

## 2. Methods

### 2.1. Participants

We recruited 53 obese healthy volunteers (31 men, 22 women, aged 18–52 years, BMI ≥ 28 kg/m^2^) from the outpatient clinic of endocrinology and metabolism department of Shanghai Jiao Tong University Affiliated Sixth People's Hospital. All the subjects underwent a general health examination before entering the study. Exclusion criteria were as follows: pregnant or plan for pregnant; lactation or postmenopausal women; use of any prescription medication in previous 2 months; had any weight loss diet or pill during the past 6 months; consuming >20 g/day of alcohol; tobacco use within 6 month; cardiovascular or endocrine disease history; hypertension history or current elevated blood pressure (systolic blood pressure (SBP): ≥150 mm Hg; diastolic blood pressure (DBP) ≥90 mm Hg; current treatment for hypertension); diabetes mellitus; acute or chronic infections; hepatopathy, kidney disease, gastrointestinal disease or any other acute or chronic diseases requiring treatment.

This study was approved by the institutional review board of Shanghai Jiao Tong University Affiliated Sixth People's Hospital in accordance with the principles of the Helsinki Declaration II. Written informed consent was obtained from each participant. There were no monetary incentives for participation.

### 2.2. Experimental Protocol

One week before initiation of the study, all subjects were asked to maintain their habitual energy intake. At baseline and after the 8-week VLCD treatment, standard 75-g oral glucose tolerance tests (OGTT) were performed. At weeks 0, 4, and 8, anthropometric parameters, bioimpedance assessments, biochemical indices, insulin resistance and sensitivity, LFP, SFA, and VFA were measured. 

### 2.3. Dietary Intervention

All subjects were given individual instructions on how to follow the VLCD. Energy intake was restricted to less than 800 kcal/day (carbohydrate intake < 20 g/d). All daily meals were replaced as follows: a cup of soybean milk (200 mL) and a boiled egg at breakfast; a diet nutrition bar (106 Kcal: 2.8 g carbohydrate, 11.2 g protein and 5.6 g fat; Nutriease Health Technology Co., Ltd., Hangzhou, China), nonstarchy vegetables (<200 kcal), and 50 g protein from meat (i.e., beef, lean pork, skinned chicken, fish) at lunch and dinner. Supplementation of multivitamins and minerals was provided per day. Subjects were also encouraged to drink at least 1.8 litres of water per day, and asked to maintain their habitual level of physical activity. Regular telephone contact to individual by nutritionists was provided for nutrient support. After the 8-week dietary intervention, there was a 1-week recovery period when the eucaloric intake was allowed to overcome the catabolic state. Then participants restarted normal eating and were provided with information about portion size and healthy eating. 

### 2.4. Measurements

#### 2.4.1. Bioimpedance Assessments

Before and after the VLCD, whole body skeletal muscle mass and fat mass were measured by a multifrequency impedance plethysmograph body composition analyzer (InBody 720, Biospace, Korea). Before analyzing, participants stand on the electrodes embedded in the scale platform of the respective analyzers and wipe the bottom of their feet with a proprietary electrolyte tissue. Then they were asked to stand upright grasping the handles of the analyzer in order to contact with 4 pairs of electrodes (octapolar technology). Resistance was measured at five specific frequencies (1 kHz, 50 kHz, 250 kHz, 500 kHz, and 1 MHz) and reactance at three specific frequencies (5 kHz, 50 kHz, and 250 kHz). Total body water (TBW) was estimated from area, volume, length, impedance, and a constant proportion (specific resistivity). Fat-free mass was estimated by dividing TBW by 0.73. After the participant's identification number, sex, age, and height were typed into the analyzer; the fat mass percentage (FMP) and skeletal muscle mass percentage of whole body weight (SMMP) were calculated by the computer software automatically [7].

#### 2.4.2. 1H Magnetic Resonance Spectroscopy (1H MRS) Measurement of Liver Fat Percent

Liver magnetic resonance imaging and in vivo single-voxel MRS were performed using Philips Achieva 3.0T MRI system (Philips Medical Systems, Eindhoven, The Netherlands) equipped with an 8-channel phase coil. Anatomical T1-weighted spin-echo MR images were obtained using the following parameters: repetition time (TR) = 550 ms; echo time (TE) = 10 ms; flip angle = 60; field of view (FOV) = 21 cm; slice thickness = 3 mm; slice spacing = 0.1 mm. ^1^H-MRS was performed for quantification analysis of metabolite concentrations in liver. Firstly, 2D-spin-echo images in the coronal and sagittal regions were obtained for image-guided localization of voxels of interest (VOI) for spectroscopic data acquisition. Secondary, single-voxel MRS was performed by a stimulated echo acquisition mode (STEAM) sequence by using the following parameters: TE = 20 ms; TR = 1,500 ms; VOX = 15 × 15 × 15 mm; total number of points = 128; total number of average = 64. Finally, eight-step phase cycling was used to suppress unwanted signals or artifacts. Signal intensities of the water peak at 4.7 p.p.m. (Sw) and the fat peak at 1.2 p.p.m. (Sf) were measured and liver fat percentage (LFP) was calculated as follows: LFP = 100 × Sf/(Sf + Sw) [8]. A LFP of 5.56% by 1H MRS is used as cutoff value for diagnosing NAFLD [9].

#### 2.4.3. Magnetic Resonance Imaging (MRI) Measurement of Abdominal Fat Areas

SFA and VFA were assessed by Philips Achieva 3.0T MRI system (Philips Medical Systems, Eindhoven, The Netherlands) using standard array coils with the subject supine. Breath-hold FISP images were centered on the L4-L5 intervertebral disc using standard localizer images with the following parameters: TR = 4 ms, TE = 2 ms, number of slices = 12, slice thickness = 8 mm, image matrix 256 × 256, and field-of-view = 500 × 500 mm. The 4 slices that were best aligned with the L4-L5 disc were analyzed by SliceOmatic 5.0 software package (Escape Medical Viewer V3.2) to define VFA and SFA [10]. VFA and SFA were measured by fitting a spline curve to points on the border of the subcutaneous and visceral regions. Nonfat regions within the visceral region were also outlined with a spline fit and subtracted from the total visceral region [11].

#### 2.4.4. Laboratory Evaluation

At baseline and week 8, all participants underwent a 75-g OGTT in the morning after 10 h overnight fasting. Their venous blood samples were drawn at 0 (fating state), 30, 60, and 120 minutes. Plasma glucose concentration was measured using a glucose oxidase method. Serum insulin levels were tested by a radioimmunoassay using the insulin detection kit, according to the manufacturer's instructions (Beijing North Institute of Biological Technology, Beijing, China). The following indices were measured by standard commercial methods on a parallel, multichannel analyzer (Hitachi 7600-020, Tokyo, Japan): liver function panel including aspartate aminotransferase (AST), alanine aminotransferase (ALT), *γ*-glutamyl transferase (*γ*-GT); renal function panel including serum creatinine, uric creatinine, and uric acid; lipid panel including total cholesterol (TC), triacylglycerol (TG), high density lipoprotein (HDL), and low-density lipoprotein cholesterol (LDL); NEFA and BHA. Urinary albumin were tested using the first-morning void urine samples by immunonephelometry on the BN II analyzer (Siemens Diagnostics) and its concentration was then corrected by urine creatinine.

### 2.5. Calculations

*β* cell function and insulin resistance were evaluated using the following formulas: (1) HOMA *β* (mIU/mmol/L) = 20 × fasting insulin (mIU/L)/(fasting glucose (mmol/L) − 3.5) [12]; (2) HOMA IR (mIU*·*mmol/L^2^) = fasting insulin (mIU/L) × fasting glucose (mmol/L)/22.5 [13]; (3) Whole Body Insulin Sensitivity Index (WBISI, L^2^/mmol^2^) = 10000/square root of [fasting glucose (mmol/L) × fasting insulin (mIU/L) × mean insulin (mIU/L) × mean glucose (mmol/L)]; mean glucose = [fasting glucose (mmol/L) + 2 h postprandial glucose (mmol/L)]/2; mean insulin = [fasting insulin (mIU/L)+ 2 h postprandial insulin (mIU/L)]/2 [14].

### 2.6. Statistical Analysis

All continuous variables were tested for normal distribution by the Kolmogorov–Smirnov normality test. Log-transformations were used to normalize skewed variables. Data were presented as mean ± SE. Chi-square test was used for categorical variables, repeated-measures ANOVA for comparison among different time points, and paired *t*-test for comparison of variables before and after the study. *P* values <0.05 (2-tailed tests) were considered to be statistically significant. Statistical analysis was performed using SPSS 17.0 (SPSS, Chicago, IL, USA).

## 3. Results

Of 53 obese subjects, 46 (86.8%) completed the study. Among the 7 participants who withdrew, one was diagnosed of type 2 diabetes, one had urine microalbumin/creatinine ≥30 ug/mg at baseline, two dropped out for stomachache, and three for noncompliance. 

### 3.1. Body Weight, Waist, and Blood Pressure

As shown in Table 1, after the 8-week VLCD intervention, from baseline, mean weight loss was −8.7 ± 0.6 kg (−9.0 ± 0.5%, *P* < 0.0001), representing a mean reduction in BMI of −3.0 ± 0.2 kg/m^2^ (−9.0 ± 0.5%, *P* < 0.0001). Mean waist circumference change was −5.9 ± 1.2 cm (from 104.5 ± 1.7 cm to 98.6 ± 1.4 cm, *P* < 0.0001), mean reductions in SBP and DBP were −13.1 ± 2.8 mmHg and −8.2 ± 1.6 mmHg respectively (all *P* values < 0.0001). 

### 3.2. Body Composition and Liver Fat Percent

**Figure 1:**
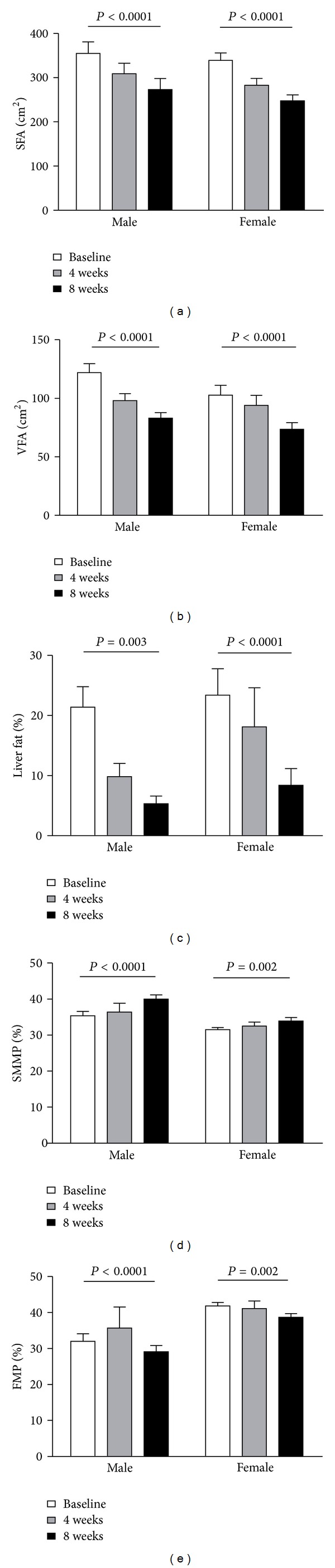
Improvement of body fat composition after an 8-week VLCD intervention. SFA: subcutaneous fat area; VFA: visceral fat area; SMMP: skeletal muscle mass percentage of whole body weight; VMP: fat mass percentage of whole body weight.

As shown in Table 2, significant decrement in mean SFA (−66.5 ± 7.9 cm^2^, *P* < 0.0001) and VFA (−35.3 ± 3.9 cm^2^, *P* < 0.0001) was observed after the 8-week VLCD diet. FMP significantly decreased (−3.0 ± 0.4%, *P* < 0.0001) while SMMP significantly increased (1.7 ± 0.2%, *P* < 0.0001). Moreover, with LFP significantly decreasing (−16.4 ± 2.4%, *P* < 0.0001), about half of the subjects who were NAFLD at baseline experienced a reversal at the end of the study (from 87.0% to 43.5%, *P* = 0.021). Furthermore, all the improvements of body composition and liver fat percent were significantly presented in both male and female subjects (Figure 1).

### 3.3. Plasma Metabolic Parameters and Hepatic Enzymes Concentrations

Together with weight loss from baseline to the end of the study, liver enzymes of ALT, AST, *γ*-GT were all significantly improved (Table 1). Concentrations of TC (from 5.12 ± 0.12 mmol/L to 4.79 ± 0.14 mmol/L, *P* < 0.0001) and TG (from 1.98 ± 0.23 mmol/L to 1.08 ± 0.09 mmol/L, *P* < 0.0001) significantly decreased and HDL significantly increased (from 1.15 ± 0.04 mmol/L to 1.19 ± 0.04, *P* = 0.01). However, the LDL level did not significantly change after the dietary intervention. The level of NEFA did not change at week 4 but decreased significantly at week 8 (baseline: 729.8 ± 34.9 *μ*mmol/L *versus* week 4: 729.3 ± 43.2  *μ*mmol/L *versus* week 8: 574.7 ± 37.0 *μ*mmol/, *P* = 0.001). Although the concentration of BHA significantly increased at week 4 (baseline: 0.06 ± 0.01 mmol/L *versus *week 4: 0.56 ± 0.13 mmol/L, *P* < 0.0001), it subsequently decreased at the end of study, with its concentration remaining significantly higher than that at baseline (baseline: 0.06 ± 0.01 mmol/L *versus *week 8: 0.24 ± 0.07 mmol/L, *P* = 0.004). As concerned with renal function, we observed the serum uric acid level significantly reduced (baseline: 395.1 ± 13.5 *μ*mol/L *versus* week 8: 369.2 ± 17.0 *μ*mol/L, *P* = 0.035), but urine microalbumin creatinine ratio (UACR) did not significantly decrease (baseline: 8.7 ± 1.0 *μ*g/mg* versus *week 8: 7.1 ± 0.7 *μ*g/mg, *P* = 0.059). 

### 3.4. Glucose Homeostasis and Insulin Sensitivity

**Figure 2:**
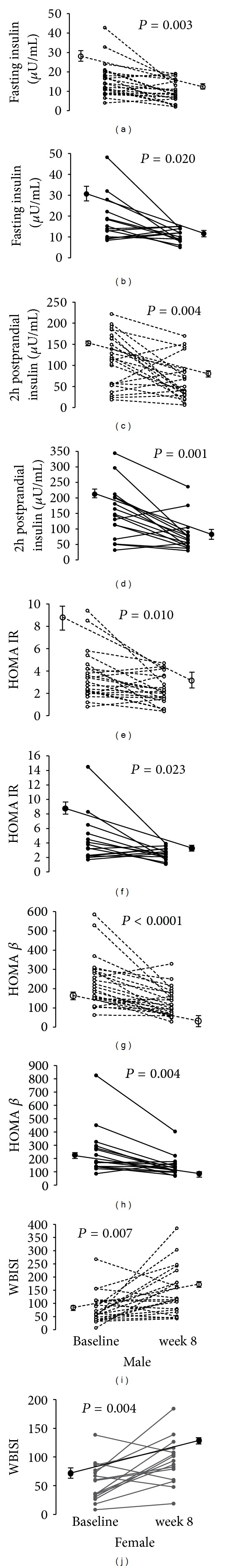
Changes of insulin resistance and sensitivity in male and female after 8 weeks of VLCD intervention. Solid lines represent the changes in male and dashed lines represent the changes in female.

As shown in Table 2, significant reductions in fasting plasma glucose (FPG), fasting insulin (FINS), and 2 h postprandial insulin (2hINS) were observed (all *P* values < 0.01). However, the change of 2 h postprandial plasma glucose (2hPG) was not statisticaly significant at the end of the study (*P* = 0.061). HOMA IR and HOMA *β* significantly improved with the reduction of −3.6 ± 1.7 and −104.8 ± 18.3 (all *P* values < 0.0001), respectively. WBISI significantly increased from 67.0 ± 6.9 to 123.7 ± 12.8 (*P* < 0.0001). Moreover, as shown in Figure 2, FINS, 2hINS, HOMA IR, HOMA *β*, and WBISI were all improved in both male and female subjects (all *P* values < 0.05). 

## 4. Discussion

The major finding in the present study was that an 8-week VLCD intervention induced a significant weight loss with reduction of abdominal subcutaneous and visceral fat mass. This hypocarbohydrate dietary also had significant effects on liver fat content reduction and improvements of metabolic parameters and insulin responsiveness. These observations suggested that a VLCD diet intake resulted in a lower insulin/glucagon ratio, which enhanced adipose tissue lipolysis and oxidation with a subsequent promotion of liver ketone bodies production for energy needs [15]. 

In our study, we only observed a significant reduction of NEFA at week 8. As a product of fat lipolysis and oxidation, BHA was in its peak level in week 4 and subsequently decreased in week 8, with its concentration remaining significantly higher than that at baseline. Recently, a study reports that NEFA significantly increased in the first 7 days but decreased in week 8 in diabetic patients who participated in an 8-week VLCD intervention [16]. Therefore, we tentatively suppose that in our study, there might also exist a similar peak of NEFA caused by lipolysis at week 1 which we did not observe. Considering the changes of NEFA and BHA together, the two level curves might reflect the process of fat burning during the 8-week period. 

It has long been presumed that weight loss after VLCD was associated with water loss [17] until Volek et al. applied dual-energy X-ray absorptiometry to examine the change of body composition in subjects who switched from their habitual diet (48% carbohydrate, 32% fat) to a VLCD (8% carbohydrate, 61% fat) for 6 weeks [18]. They surprisingly found that fat mass significantly decreased and lean body mass significantly increased, despite no change in physical activity. Our results were similar to their findings that SMMP significantly increased and FMP significantly decreased at the end of the dietary intervention. It was suggested that water may account for some of the initial rapid weight loss, but fat loss subsequently accelerated and lean tissue was preserved over a longer period, which may be the main cause of weight loss [19].

After the 8-week dietary intervention, with the LFP significantly decreasing, half of the obese subjects with NAFLD had a reversal. Meanwhile, significant improvements of ALT, AST, and *γ*-GT were observed. Additionally, significant reduction of TC and TG and increment of HDL may have beneficial effects on the prevention of cardiovascular disease.

Several intervention studies and meta-analyses, varying in length from days to months have explored the impacts of VLCD diet on *β* cell function and insulin sensitivity [2, 4, 20, 21]. Most of the studies found that insulin sensitivity improved together with weight loss. In the present study, we found the similarities that HOMA IR, HOMA *β*, and WBISI were all significantly improved. There were several mechanisms involved in the amelioration of *β* cell function and insulin sensitivity. One of the major hypothesis was that the reduction of triacylglycerol stores in pancreas and liver contributed to the normalization of *β* cell function [16]. Furthermore, decreasing of triacylglycerol in skeletal muscle may be another cause of hepatic resistance alleviation [22]. However, the results from several studies demonstrated that insulin sensitivity did not alter after a short-term VLCD intervention for 5–7 days [4, 23]. It might be a too short intervention period to detect the changes in insulin sensitivity [23].

In order to observe whether the effects of a VLCD were similar in different gender, we further analysed the body composition and insulin sensitivity in male and female, respectively. The results presented a similar beneficial effects in both genders. 

The present study has some limitations. Firstly, we did not measure the metabolic parameters at the first week which may contribute to interpret the acute effect of VLCD. Secondly, for the lacking measurement of intramuscular triacylglycerol content, the relation between the change of it and improvement of insulin sensitivity was unclear. Finally, being a short-term designed study, the evidence of the longer effects of VLCD was unclear. So longer-term and further studies are needed to be designed to investigate the effects and to reveal the underlying mechanisms of VLCD in the future.

In conclusion, in both male and female, rapid weight reduction induced by an 8-week VLCD intervention effectively decreased total abdominal subcutaneous and visceral adipose tissue compartments, and liver fat content, increased skeletal muscle percentage of whole body weight, improved metabolic profile, and insulin resistance and sensitivity. All these beneficial effects might have been due to enhanced hepatic and whole-body lipolysis and oxidation.

## Figures and Tables

**Table 1:** Effects of anthropometric and biochemical parameters in obese subjects after a VLCD intervention.

	Baseline	Week 4	Week 8	Change from baseline to Week 8	*P *
Weight, Kg	96.1 ± 2.7	90.1 ± 2.6*	87.4 ± 2.5*	−8.7 ± 0.6	<0.0001
BMI, Kg/m^2^	32.6 ± 0.6	30.6 ± 0.6*	29.7 ± 0.6*	−3.0 ± 0.2	<0.0001
Waist, cm	104.5 ± 1.7	100.7 ± 1.5*	98.6 ± 1.4*	−5.9 ± 1.2	<0.0001
SBP, mmHg	135.2 ± 2.8	124.1 ± 2.0*	121.7 ± 2.0*	−13.1 ± 2.8	<0.0001
DBP, mmHg	82.7 ± 1.7	76.7 ± 1.5*	73.9 ± 1.6*	−8.2 ± 1.6	<0.0001
ALT, U/L	42.9 ± 5.1	31.6 ± 3.5*	22.3 ± 1.6*	−20.6 ± 5.2	0.002
AST, U/L	26.2 ± 1.9	23.0 ± 1.5**	20.3 ± 1.5*	−6.0 ± 1.7	0.001
*γ*-GT, U/L	55.1 ± 6.5	27.2 ± 2.4*	27.8 ± 3.5*	−30.4 ± 6.3	<0.0001
Serum uric acid, *μ*mol/L	395.1 ± 13.5	382.5 ± 14.2	369.2 ± 17.0**	−26.1 ± 10.0	0.035
UACR, *μ*g/mg	8.7 ± 1.0	7.5 ± 0.9	7.1 ± 0.7	−1.9 ± 0.9	0.059
TC, mmol/L	5.12 ± 0.12	4.60 ± 0.13*	4.79 ± 0.14*	−0.37 ± 0.14	<0.0001
TG, mmol/L	1.98 ± 0.23	1.11 ± 0.10*	1.08 ± 0.09*	−0.98 ± 0.23	<0.0001
HDL, mmol/L	1.15 ± 0.04	1.09 ± 0.03**	1.19 ± 0.04	0.02 ± 0.03	0.01
LDL, mmol/L	3.09 ± 0.12	3.01 ± 0.12	3.13 ± 0.13	0.05 ± 0.10	0.407
NEFA, *μ*mmol/L	729.8 ± 34.9	729.3 ± 43.2	574.7 ± 37.0*	−166.2 ± 47.6	0.001
BHA, mmol/L	0.06 ± 0.01	0.56 ± 0.13*	0.24 ± 0.07*	0.15 ± 0.06	0.004

Data are presented as mean ± standard error (SE). BMI: body mass index; SBP: systolic blood pressure; DBP: systolic bold pressure; TC: total cholesterol; TG: triacylglycerol; HDL: high density lipoprotein; LDL: low density lipoprotein; NEFA: non-esterified fatty acid; BHA: *β*-hydroxybutyric acid; ALT: alanine aminotransferase; AST: aspartate aminotransferase; *γ*-GT: *γ*-glutamyl transferase; UACR: urine microalbumin creatinine ratio. *P*: changes of sequential data within experiments from baseline to week 8. Significant difference observed versus baseline: *P < 0.001; **P < 0.05.

**Table 2:** Effects of fat distribution, glucose homeostasis, and insulin sensitivity in obese subjects after a VLCD intervention.

	Baseline	Week 4	Week 8	Change from baseline to week 8	*P *
SFA, cm^2^	348.0 ± 16.7	299.4 ± 16.2	264.0 ± 16.4	−66.5 ± 7.9	<0.0001
VFA, cm^2^	113.9 ± 5.8	96.6 ± 4.9	79.8 ± 3.7	−35.3 ± 3.9	<0.0001
Liver fat, %	22.3 ± 2.7	13.5 ± 3.2	6.5 ± 1.4	−16.4 ± 2.4	<0.0001
SMMP, %	33.7 ± 0.9	34.1 ± 1.3	36.7 ± 0.8	1.7 ± 0.2	<0.0001
FMP, %	37.2 ± 1.4	38.9 ± 2.0	34.3 ± 1.3	−3.0 ± 0.4	<0.0001
FPG, mmol/L	5.3 ± 0.1	5.0 ± 0.1	5.2 ± 0.1	−0.07 ± 0.15	0.001
2h PG, mmol/L	7.5 ± 0.3	—	6.9 ± 0.3	−0.59 ± 0.31	0.061
FINS, *μ*U/mL	22.5 ± 4.0	10.2 ± 0.8	11.8 ± 1.4	−11.5 ± 4.3	0.003
2h INS, *μ*U/mL	134.2 ± 11.3	—	71.1 ± 8.2	−57.2 ± 11.3	<0.0001
HOMA IR	6.1 ± 1.5	2.3 ± 0.2	2.8 ± 0.4	−3.6 ± 1.7	0.006
HOMA *β*	235.7 ± 21.6	140.3 ± 11.4	136.2 ± 12.1	−104.8 ± 18.3	<0.0001
WBISI	67.0 ± 6.9	—	123.7 ± 12.8	54.2 ± 13.9	<0.0001

Data are presented as mean ± standard error (SE). SFA: subcutaneous fat area; VFA: visceral fat area; SMMP: skeletal muscle mass percentage of whole body weight; FMP: fat mass percentage of whole body weight;. FPG: fasting plasma glucose; 2hPG: 2h postprandial glucose; FINS: fasting insulin; WBISI: whole body insulin sensitivity index. *P*: changes of sequential data within experiments from baseline to week 8. Significant difference observed versus baseline: *P < 0.001; **P < 0.05.
